# Seasonal Dynamics of Epiphytic Microbial Communities on Marine Macrophyte Surfaces

**DOI:** 10.3389/fmicb.2021.671342

**Published:** 2021-09-09

**Authors:** Marino Korlević, Marsej Markovski, Zihao Zhao, Gerhard J. Herndl, Mirjana Najdek

**Affiliations:** ^1^Center for Marine Research, Ruđer Bošković Institute, Rovinj, Croatia; ^2^Department of Functional and Evolutionary Ecology, University of Vienna, Vienna, Austria; ^3^Department of Marine Microbiology and Biogeochemistry, Royal Netherlands Institute for Sea Research (NIOZ), Utrecht University, Den Burg, Netherlands

**Keywords:** epiphytic microbial community, seasonal dynamics, Illumina 16S rRNA sequencing, *Cymodocea nodosa*, *Caulerpa cylindracea*

## Abstract

Surfaces of marine macrophytes are inhabited by diverse microbial communities. Most studies focusing on epiphytic communities of macrophytes did not take into account temporal changes or applied low sampling frequency approaches. The seasonal dynamics of epiphytic microbial communities was determined in a meadow of *Cymodocea nodosa* invaded by *Caulerpa cylindracea* and in a monospecific settlement of *C. cylindracea* at monthly intervals. For comparison the ambient prokaryotic picoplankton community was also characterized. At the OTU level, the microbial community composition differed between the ambient water and the epiphytic communities exhibiting host-specificity. Also, successional changes were observed connected to the macrophyte growth cycle. Taxonomic analysis, however, showed similar high rank taxa (phyla and classes) in the ambient water and the epiphytic communities, with the exception of *Desulfobacterota*, which were only found on *C. cylindracea*. *Cyanobacteria* showed seasonal changes while other high rank taxa were present throughout the year. In months of high *Cyanobacteria* presence the majority of cyanobacterial sequences were classified as *Pleurocapsa*. Phylogenetic groups present throughout the year (e.g., *Saprospiraceae, Rhodobacteraceae*, members without known relatives within *Gammaproteobacteria, Desulfatitalea*, and members without known relatives within *Desulfocapsaceae*) constituted most of the sequences, while less abundant taxa showed seasonal patterns connected to the macrophyte growth cycle. Taken together, epiphytic microbial communities of the seagrass *C. nodosa* and the macroalga *C. cylindracea* appear to be host-specific and contain taxa that undergo successional changes.

## 1. Introduction

Marine macrophytes (seagrasses and macroalgae) are important ecosystem engineers forming close associations with microorganisms belonging to all three domains of life (Egan et al., 2013; Tarquinio et al., 2019). Microbes can live within macrophyte tissue as endophytes or form epiphytic communities on surfaces of leaves, roots, rhizomes, and thalli (Egan et al., 2013; Hollants et al., 2013; Aires et al., 2015; Tarquinio et al., 2019). Epiphytic and endophytic microbial communities exhibit a close functional relationship with the macrophyte host. It has been proposed that this close relationship constitutes a holobiont, an integrated community where the macrophyte organism and its symbiotic partners support each other (Margulis, 1991). In addition, as suggested by the hologenome theory endophytic microbes play a critical role in the adaptation and evolution of the host species (Aires et al., 2015).

Biofilms of microbial epiphytes can contain diverse taxonomic groups and harbor cell abundances from 10^2^ to 10^7^ cells cm-2 (Armstrong et al., 2000; Bengtsson et al., 2010; Burke et al., 2011b). In such an environment a number of positive and negative interactions between the macrophyte and the colonizing microorganisms have been described (Egan et al., 2013; Hollants et al., 2013; Tarquinio et al., 2019). Macrophytes can promote growth of associated microbes by nutrient exudation (Wood and Hayasaka, 1981), while in return microorganisms may support macrophyte performance through improved nutrient availability (Nielsen et al., 2001; de Oliveira et al., 2012), phytohormone production (Matsuo et al., 2003; Celdran et al., 2012), and protection from toxic compounds (Küsel et al., 2006), oxidative stress (Sanchez-Amat et al., 2010), biofouling organisms (Dobretsov and Qian, 2002), and pathogens (Penesyan et al., 2009). Besides these positive interactions, macrophytes can negatively impact the associated microbes by producing reactive oxygen species (Weinberger, 2007) and secondary metabolites (Saha et al., 2011).

All these ecological roles are carried out by a taxonomically diverse community of microorganisms. At higher taxonomic ranks (phyla and classes) microbial taxa, such as *Alphaproteobacteria, Gammaproteobacteria, Bacteroidota*, and *Cyanobacteria*, have been associated with surfaces of seagrass leaves and macroalgal thalli (Crump and Koch, 2008; Tujula et al., 2010; Lachnit et al., 2011; Egan et al., 2013; Tarquinio et al., 2019; Ugarelli et al., 2019). While similar high rank taxa have been found on surfaces of different macrophyte species, in order to describe new ecological patterns it is also necessary to focus on lower taxonomic ranks (genus and OTUs) which tend to be host-specific (Lachnit et al., 2011; Hollants et al., 2013; Roth-Schulze et al., 2016). While the microbial community composition can vary between host species, metagenomic analyses revealed that the majority of microbial functions are conserved, showing that different epiphytic microbial species could be functionally similar (Burke et al., 2011a; Roth-Schulze et al., 2016; Cúcio et al., 2018). This discrepancy between the microbial taxonomic and functional composition might be explained by the lottery hypothesis (Sale, 1976). It postulates that an initial random colonization step takes place from a set of functionally equivalent taxonomic groups resulting in taxonomically different epiphytic communities sharing a core set of functional genes (Burke et al., 2011a; Stratil et al., 2013; Schmidt et al., 2015; Roth-Schulze et al., 2016).

Seagrasses are known to form close relationships with microbial communities associated with the surfaces of leaves, roots, and rhizomes (Cúcio et al., 2016; Crump et al., 2018; Ugarelli et al., 2019; Ettinger and Eisen, 2020; Wang et al., 2020). For different seagrass species a distinct microbial community from ambient seawater or bulk sediment has been reported, however no species specific communities have been found (Cúcio et al., 2016; Crump et al., 2018; Ugarelli et al., 2019). It seems that seagrasses are selecting the associated microbial community but these microbes have not coevolved with their seagrass plant host. Similar to seagrasses, siphonous macroalgae of the genus *Caulerpa* are also closely associated with their microbial communities (Aires et al., 2013, 2015; Rizzo et al., 2016b; Stabili et al., 2017; Morrissey et al., 2019). While some studies have found similar culturable bacterial groups associated with the surface of a *Caulerpa* species from different geographic locations (Stabili et al., 2017), other have reported large compositional differences that were mainly attributed to different host species of this genus, biogeography, and nutrient levels (Morrissey et al., 2019) again raising the question to which extent are associated communities host-specific.

Since marine macrophytes in temperate zones are exhibiting seasonal changes in growth and physiology (Agostini et al., 2003; Najdek et al., 2020) it is important to verify if and how surface associated microbial communities are affected by these changes. The majority of studies describing macrophyte epiphytic microbial communities have not included possible seasonal changes (Crump and Koch, 2008; Lachnit et al., 2009; Burke et al., 2011b; Roth-Schulze et al., 2016; Ugarelli et al., 2019). If seasonal changes have been taken into account, low temporal frequency, applied methodologies, and/or limited number of analyzed host species did not allow for a detailed taxonomic analysis (Tujula et al., 2010; Lachnit et al., 2011; Bengtsson et al., 2012; Michelou et al., 2013; Miranda et al., 2013; Mancuso et al., 2016). In the present study we performed a descriptive analysis of seasonal bacterial and archeal community dynamics on the surfaces of the seagrass *Cymodocea nodosa*, an abundant seagrass species in the Mediterranean (Short et al., 2001), and siphonous macroalga *Caulerpa cylindracea*, one of the most invasive macroalgal species (Klein and Verlaque, 2008; Boudouresque et al., 2009). Bacterial and archeal epiphytes were sampled in a meadow of *C. nodosa* invaded by the invasive *C. cylindracea* and in a locality of only *C. cylindracea* located in the proximity of the seagrass meadow. For comparison, the microbial community of the ambient seawater was also characterized. The presence of both macrophytes in the same area enabled (i) the assessment of differences in the bacterial and archeal communities between host species and settlements of *C. cylindracea* and (ii) the evaluation of differences between surface associated and free living (ambient seawater) communities. In addition, these differences were evaluated on a monthly scale providing insight into seasonal changes (iii).

## 2. Materials and Methods

### 2.1. Sampling

**Figure 1:**
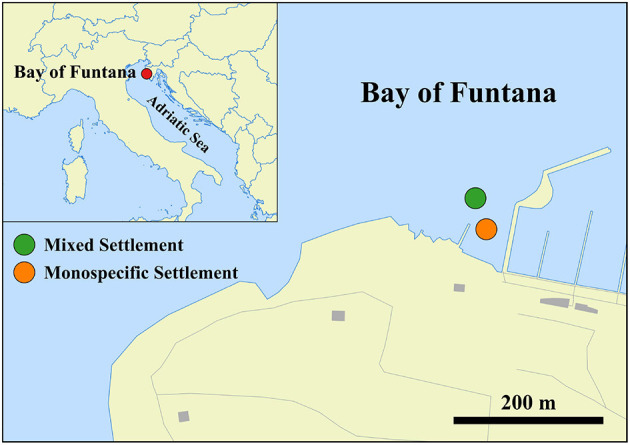
Location of the mixed (*C. nodosa* and *C. cylindracea*) and monospecific (*C. cylindracea*) settlement in the Bay of Funtana, northern Adriatic Sea (© OpenStreetMap contributors, www.openstreetmap.org/copyright).

Sampling was performed in the Bay of Funtana, northern Adriatic Sea (45°10′39′′ N, 13°35′42′′ E). The sea floor in the bay is partly covered by the invasive macroalga *C. cylindracea* that can be found in a monospecific settlement or mixed with the seagrass *C. nodosa* (Figure 1). *C. nodosa* leaves were retrieved from a meadow of *C. nodosa* invaded by the invasive *C. cylindracea* (mixed settlement; depth, 2–2.5 m), while *C. cylindracea* thalli were sampled in the same invaded meadow (mixed settlement; depth, 2–2.5 m) and in a monospecific settlement (depth, 1–1.5 m) of *C. cylindracea* located in the proximity (20–50 m) of the invaded meadow at approximately monthly intervals from November 2017 to October 2018 (Supplementary Table 1). Leaves and thalli were collected by diving and transported to the laboratory in containers placed on ice and filled with seawater collected at the sampling site. Upon arrival to the laboratory, *C. nodosa* leaves were cut into sections of 1–2 cm, while *C. cylindracea* thalli were cut into 5–8 cm long sections. Leaves and thalli were washed three times with sterile artificial seawater (ASW) to remove loosely attached microbial cells. Ambient seawater was collected in 10 L containers by diving and transported to the laboratory where 10–20 L were filtered through a 20 μm net. The filtrate was further sequentially filtered through 3 and 0.2 μm polycarbonate membrane filters (Whatman, United Kingdom) using a peristaltic pump. Filters were briefly dried at room temperature and stored at -80°C. Seawater samples were also collected approximately monthly from July 2017 to October 2018.

### 2.2. DNA Isolation

DNA from surfaces of *C. nodosa* and *C. cylindracea* was isolated from a pool of leaves (1 g wet weight) or thalli (2 g wet weight) on the sampling day using a previously modified and adapted protocol that allows for a selective epiphytic DNA isolation (Massana et al., 1997; Korlević et al., 2021). Briefly, leaves and thalli were incubated in a lysis buffer and treated with lysozyme and proteinase K. Following the incubations, the mixture containing lysed epiphytic cells was separated from the leaves and thalli and extracted using phenol-chloroform. Finally, the extracted DNA was precipitated using isopropanol. DNA from seawater picoplankton was extracted from 0.2 μm polycarbonate filters according to Massana et al. (1997) with a slight modification. Following the phenol-chloroform extraction, 1/10 of 3 m sodium acetate (pH 5.2) was added. DNA was precipitated by adding 1 volume of chilled isopropanol, incubating the mixtures overnight at -20°C and centrifuging at 20,000 × *g* and 4°C for 20 min. The pellet was washed twice with 500 μl of chilled 70% ethanol and centrifuged after each washing step at 20,000 × and 4°C for 5 min. Dried pellets were re-suspended in 50–100 μl of deionized water. One DNA sample originating from seawater picoplankton was obtained per each sampling point.

### 2.3. Illumina 16S rRNA Sequencing

Illumina MiSeq sequencing of the V4 region of the 16S rRNA gene was performed as described previously (Korlević et al., 2021). The V4 region of the 16S rRNA gene was amplified using a two-step PCR procedure. In the first PCR, the 515F (5′-GTGYCAGCMGCCGCGGTAA-3′) and 806R (5′-GGACTACNVGGGTWTCTAAT-3′) primers from the Earth Microbiome Project (https://earthmicrobiome.org/protocols-and-standards/16s/) were used (Caporaso et al., 2012; Apprill et al., 2015; Parada et al., 2016). These primers contained on their 5′ end a tagged sequence. Purified PCR products were sent for Illumina MiSeq sequencing at IMGM Laboratories, Martinsried, Germany. Prior to sequencing at IMGM, the second PCR amplification of the two-step PCR procedure was performed using primers targeting the tagged region incorporated in the first PCR. In addition, these primers contained adapter and sample-specific index sequences. Beside samples, a positive and negative control for each sequencing batch was sequenced. The negative control comprised PCR reactions without DNA template, while for a positive control a mock community composed of evenly mixed DNA material originating from 20 bacterial strains (ATCC MSA-1002, ATCC, USA) was used. Sequences obtained in this study have been deposited in the European Nucleotide Archive (ENA) at EMBL-EBI under the accession number PRJEB37267 (https://www.ebi.ac.uk/ena/browser/view/PRJEB37267).

### 2.4. Sequence and Data Analysis

Obtained sequences were analyzed on the computer cluster Isabella (University Computing Center, University of Zagreb) using mothur (version 1.43.0; Schloss et al., 2009) according to the MiSeq Standard Operating Procedure (MiSeq SOP; https://mothur.org/wiki/MiSeq_SOP; Kozich et al., 2013) and recommendations provided by the Riffomonas project to enhance data reproducibility (http://www.riffomonas.org/). For alignment and classification of sequences the SILVA SSU Ref NR 99 database (release 138; http://www.arb-silva.de) was used (Quast et al., 2013; Yilmaz et al., 2014). Sequences were clustered into operational taxonomic units (OTUs) at a similarity level of 97%.

Pipeline data processing and visualization was done using R (version 3.6.0) (R Core Team, 2019) combined with packages vegan (version 2.5-6) (Oksanen et al., 2019), tidyverse (version 1.3.0) (Wickham, 2017; Wickham et al., 2019), and multiple other packages (Neuwirth, 2014; Xie, 2014, 2015, 2019a,b,c; Xie et al., 2018; Allaire et al., 2019; Wilke, 2019; Zhu, 2019). Observed number of OTUs, Chao1, ACE, exponential of the Shannon diversity index and Inverse Simpson index were calculated after normalization to the minimum number of reads per sample using vegan's function rrarefy to account for different sequencing depths (Oksanen et al., 2019). Chao1 and ACE estimators were calculated using vegan's function estimateR, while Shannon and Inverse Simpson diversity indices were retrieved using vegan's function diversity (Oksanen et al., 2019). To express both diversity indices in terms of effective number of OTUs the exponential of the Shannon diversity index was calculated (Jost, 2006). The proportions of shared OTUs and communities between samples and community types (seawater, *C. nodosa* [mixed], *C. cylindracea* [mixed] and *C. cylindracea* [monospecific]) were expressed as the Jaccard's (on presence/absence data) and Bray-Curtis similarity coefficient, respectively. The coefficients were calculated on the OTU data table using vegan's function vegdist and converted from dissimilarities to similarities (Borcard et al., 2011; Legendre and Legendre, 2012; Oksanen et al., 2019). The Principal Coordinates Analysis (PCoA) was performed on Bray-Curtis dissimilarities based on OTU abundances using the function cmdscale (Borcard et al., 2011; Legendre and Legendre, 2012). Differences between communities were tested by performing the Analysis of Similarities (ANOSIM) using the vegan's function anosim and 1,000 permutations (Oksanen et al., 2019), while differences in relative contributions or proportions of shared OTUs and communities were tested by applying the Mann–Whitney *U*-test using the function wilcox.test. In addition, differences between community type estimators or indices were tested by performing the Kruskal-Wallis *H*-test (function kruskal.test) followed by a pairwise comparison using the Mann-Whitney *U*-test (function pairwise.wilcox). Bonferroni correction was used to address the problem of multiple comparisons.

A total of 1.7 million sequences after quality curation and exclusion of sequences without known relatives (no relative sequences) and eukaryotic, chloroplast, and mitochondrial sequences were obtained (Supplementary Table 1). The number of reads per sample ranged between 8,408 and 77,463 sequences (Supplementary Table 1). Even when the highest sequencing effort was applied the rarefaction curves did not level off as commonly observed in high-throughput 16S rRNA amplicon sequencing approaches (Supplementary Figure 1). Following quality curation and exclusion of sequences as mentioned above reads were clustered into 28,750 different OTUs. Read numbers were normalized to the minimum number of sequences (8,408, Supplementary Table 1) using previously mentioned vegan's function rrarefy resulting in 17,201 different OTUs ranging from 352 to 2,062 OTUs per sample (Supplementary Figure 2). Based on the ATCC MSA-1002 mock community included in the analysis an average sequencing error rate of 0.01% was determined, which is in line with previously reported values for next-generation sequencing data (Kozich et al., 2013; Schloss et al., 2016). In addition, the negative controls processed together with the samples yielded only two sequences after sequence quality curation. The detailed analysis procedure including the R Markdown file is available as a GitHub repository (https://github.com/MicrobesRovinj/Korlevic_EpiphyticDynamics_FrontMicrobiol_2021).

## 3. Results

A total of 35 samples originating from epiphytic archeal and bacterial communities associated with surfaces of the seagrass *C. nodosa* and the macroalga *C. cylindracea* were analyzed. In addition, 18 samples (one of the samples was sequenced twice) originating from the ambient seawater were also processed for comparison. Generally, richness estimators and diversity indices showed similar trends. On average, higher values were found for *C. cylindracea* (mixed [Number of OTUs, 1,688.4 ± 136.6 OTUs] and monospecific [Number of OTUs, 1,750.4 ± 165.7 OTUs]) than for *C. nodosa* (Number of OTUs, 1,063.7 ± 210.6 OTUs) and lowest values were obtained for the microbial community of the ambient seawater (Number of OTUs, 531.0 ± 143.9 OTUs; Kruskal-Wallis, *p* < 0.0001) (Supplementary Figure 2 and Supplementary Tables 2, 3). Temporal changes did not reveal such large dissimilarities. *C. nodosa* communities showed a slow increase in all calculated richness estimators toward the end of the study, while *C. cylindracea* (mixed and monospecific) communities were characterized by slightly higher values in spring and summer than in autumn and winter (Supplementary Figure 2).

**Figure 2:**
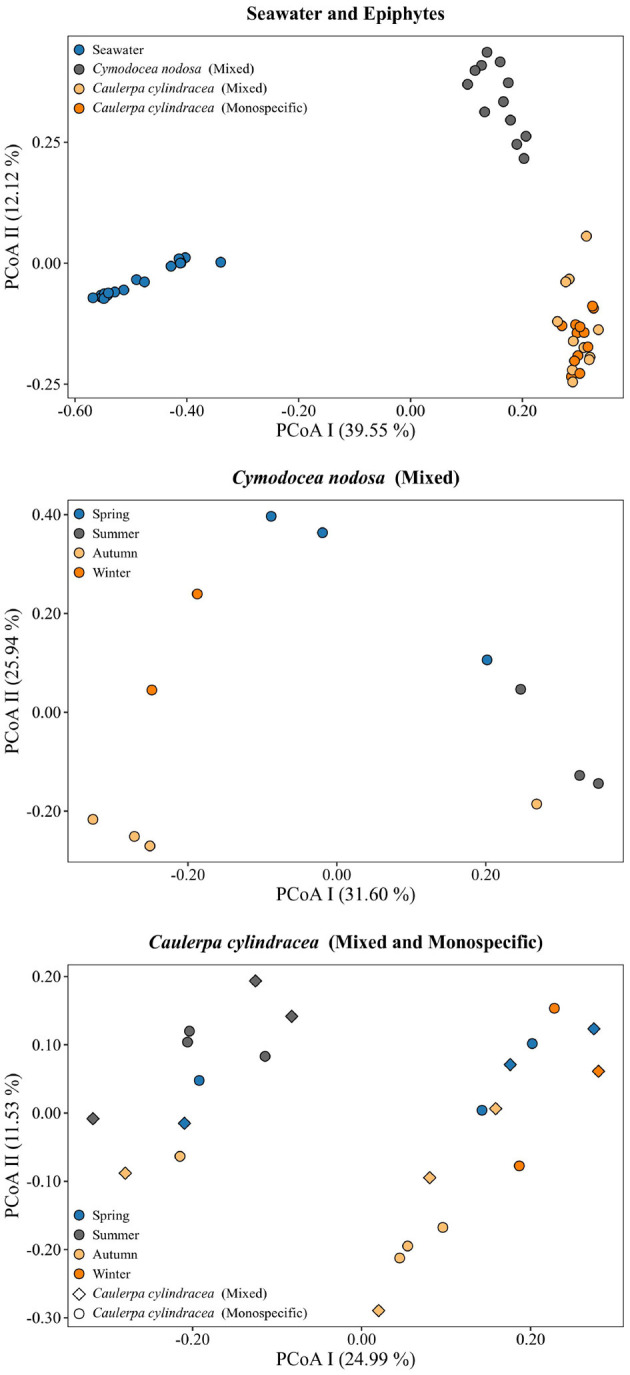
Principal Coordinates Analysis (PCoA) of Bray-Curtis distances based on OTU abundances of bacterial and archeal communities from the surfaces of the macrophytes *C. nodosa* (mixed settlement) and *C. cylindracea* (mixed and monospecific settlement) and in the ambient seawater. Samples from different environments or seasons are labeled in different color and shape. The proportion of explained variation by each axis is shown on the corresponding axis in parentheses.

**Figure 3:**
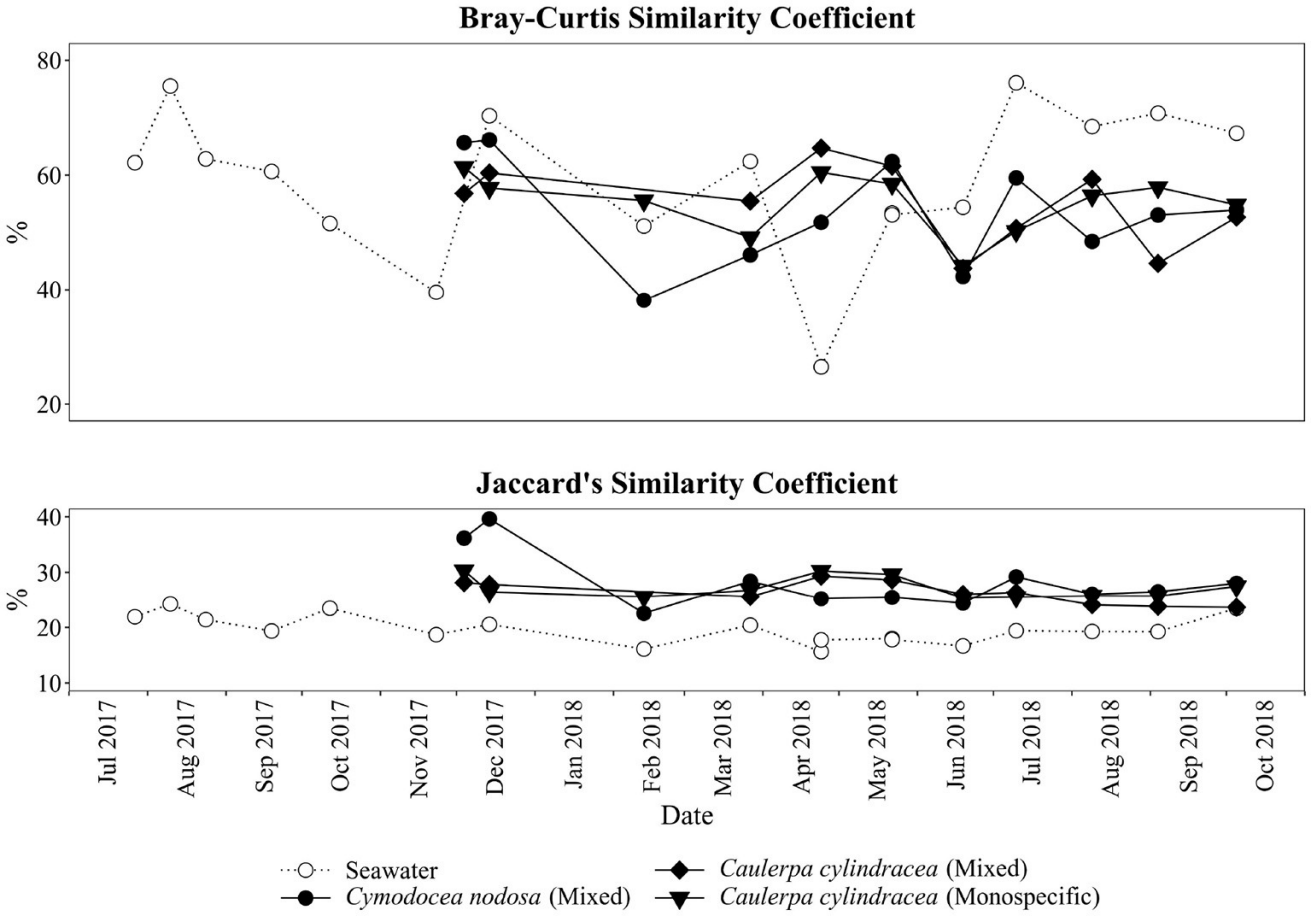
Proportion of shared bacterial and archeal communities (Bray-Curtis similarity coefficient) and shared bacterial and archaeal OTUs (Jaccard's similarity coefficient) between consecutive sampling dates and from the surfaces of the macrophytes *C. nodosa* (mixed settlement) and *C. cylindracea* (mixed and monospecific settlement) and in the ambient seawater.

A clear separation between ambient seawater and surface associated communities was found (Figure 2). In addition, a separation of epiphytic bacterial and archeal communities based on host species was detected. This separation was further supported by ANOSIM (*R* = 0.96, *p* < 0.001). The highest proportion of shared OTUs and community was found between mixed and monospecific *C. cylindracea* (Jaccard, 0.35; Bray-Curtis, 0.77), while lower shared values were calculated between ambient seawater and epiphytic communities (Jaccard, 0.10–0.11; Bray-Curtis, 0.05–0.06). Shared proportions of OTUs and communities between *C. nodosa* and *C. cylindracea* (either mixed or monospecific) were approximately in-between the values obtained for the comparison of ambient seawater with all other communities and for the comparison of the mixed and monospecific *C. cylindracea* associated community. Seasonal changes of *C. nodosa* associated communities indicated a separation between spring, summer, and autumn/winter samples (ANOSIM, *R* = 0.56, *p* < 0.01). For *C. cylindracea* associated communities a separation between summer and autumn/winter/spring samples was observed that was, however, not as strong as for *C. nodosa* associated communities (ANOSIM, *R* = 0.30, *p* < 0.05; Figure 2). Shared proportions of OTUs between consecutive sampling points were lower for ambient seawater (19.6 ± 2.5%) than for *C. nodosa* (28.3 ± 5.2%) and *C. cylindracea* (mixed [26.3 ± 2.1%] and monospecific [27.2 ± 2.0%]) associated communities (*p* < 0.0001), while mean proportions of shared communities between consecutive sampling points did not show such a difference (seawater, 57.4 ± 14.7%; *C. nodosa*, 53.4 ± 9.3%; *C. cylindracea* [mixed], 55.0 ± 7.0%; *C. cylindracea* [monospecific], 55.1 ± 5.2%; *p* = 0.1), although in ambient seawater higher fluctuations could be observed (Figure 3). In addition, only 0.4–1.0% of OTUs from each surface associated community were present at all seasons. These persistent OTUs constituted a high proportion of total sequences (40.2–53.2 %) and were mainly contributing to abundant phylogenetic groups present throughout the year, e.g. the no relative *Rhodobacteraceae* in the case of *C. nodosa* or taxa within *Desulfobacterota* in the case of *C. cylindracea* (see below; Supplementary Table 4).

**Figure 4:**
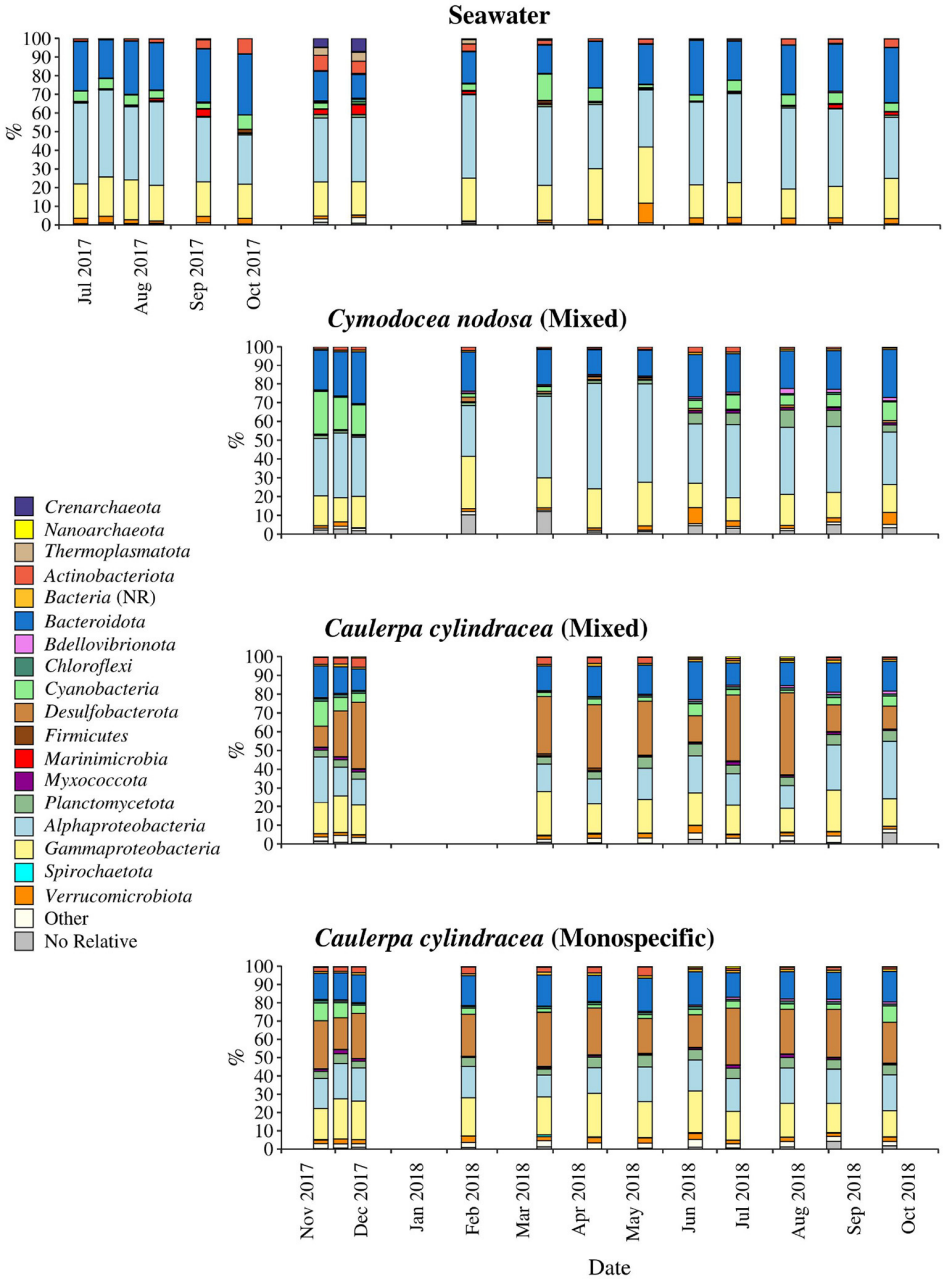
Taxonomic classification and relative contribution of the most abundant (≥1%) bacterial and archeal sequences on the surfaces of the macrophytes *C. nodosa* (mixed settlement) and *C. cylindracea* (mixed and monospecific settlement) and in the ambient seawater. NR, no relative (sequences without known relatives within the corresponding group).

**Figure 5:**
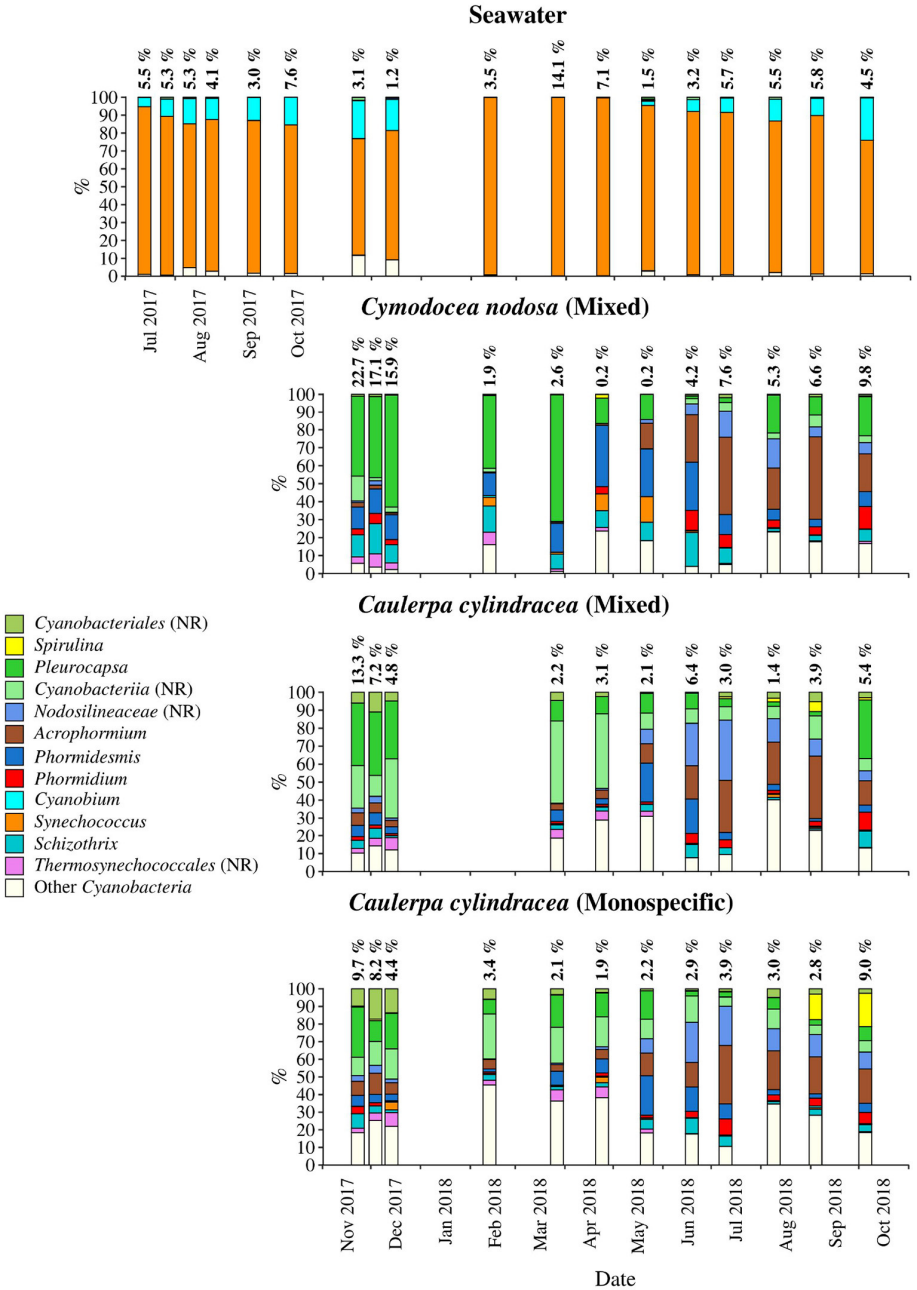
Taxonomic classification and relative contribution of the most abundant (≥1%) cyanobacterial sequences on the surfaces of the macrophytes *C. nodosa* (mixed settlement) and *C. cylindracea* (mixed and monospecific settlement) and in the ambient seawater. The proportion of cyanobacterial sequences in the total bacterial and archeal community is given above the corresponding bar. NR, no relative (sequences without known relatives within the corresponding group).

**Figure 6:**
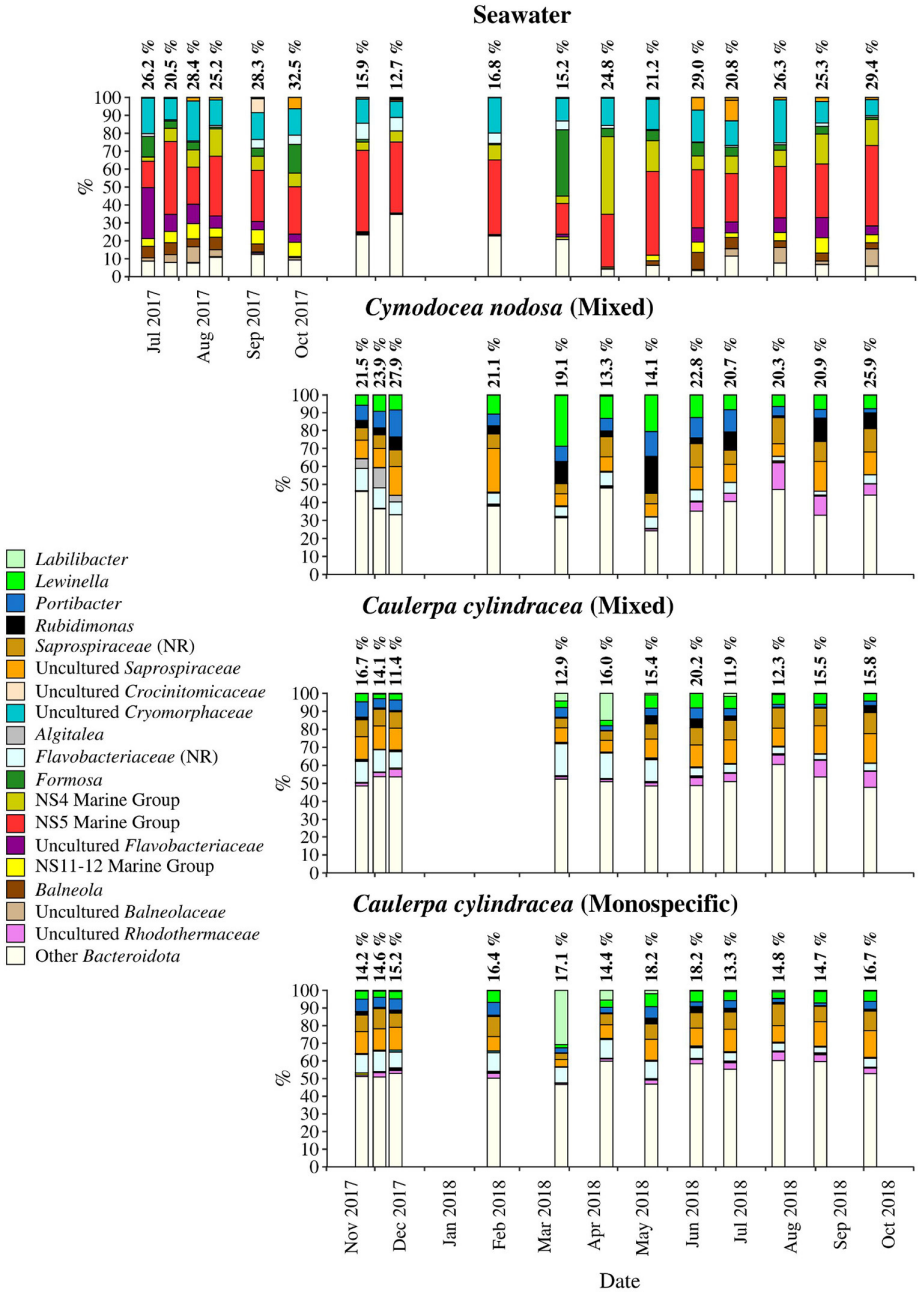
Taxonomic classification and relative contribution of the most abundant (≥ 2%) sequences within the *Bacteroidota* on the surfaces of the macrophytes *C. nodosa* (mixed settlement) and *C. cylindracea* (mixed and monospecific settlement) and in the ambient seawater. The proportion of sequences classified as *Bacteroidota* in the total bacterial and archaeal community is given above the corresponding bar. NR – No Relative (sequences without known relatives within the corresponding group).

**Figure 7:**
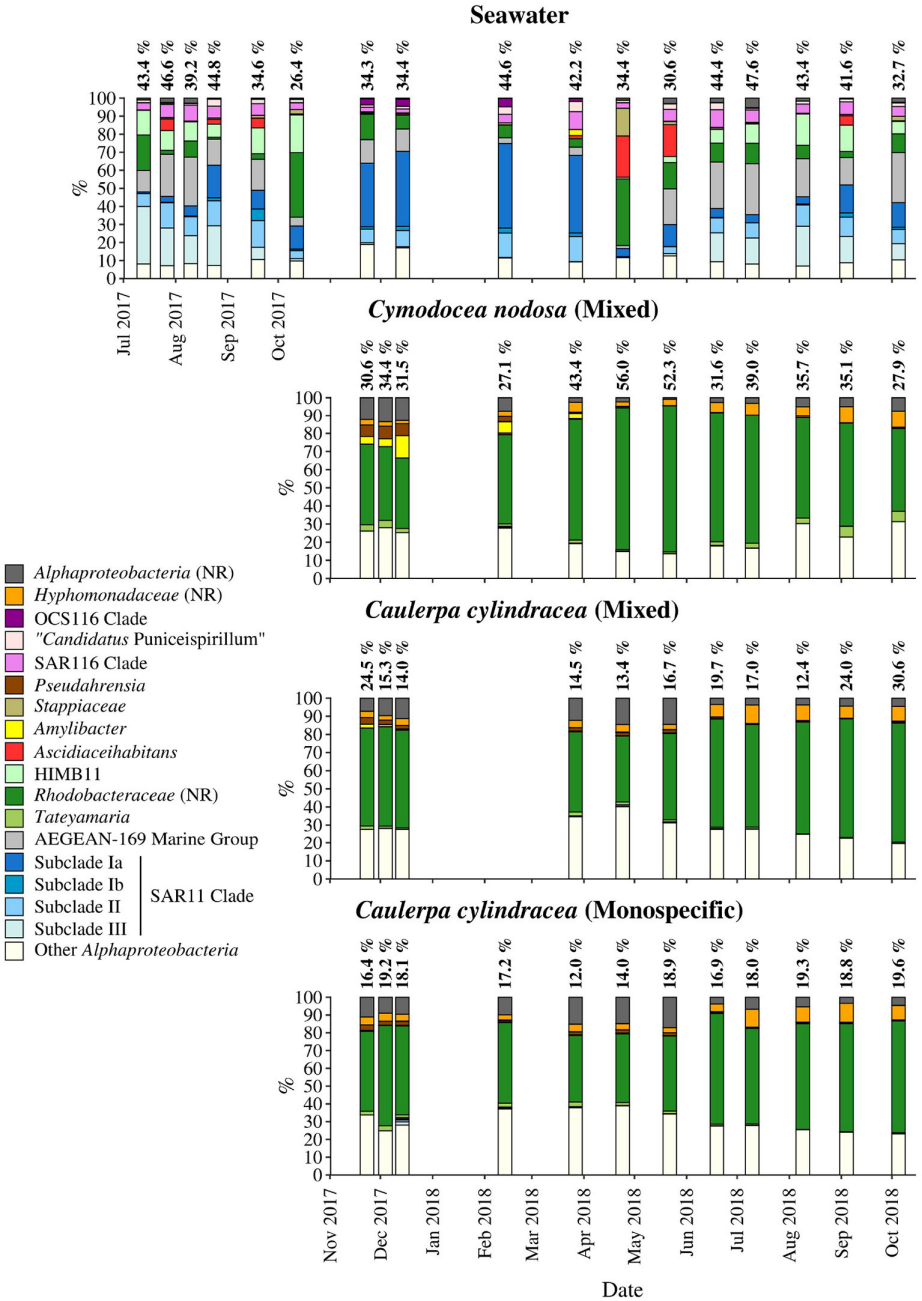
Taxonomic classification and relative contribution of the most abundant (≥ 2%) alphaproteobacterial sequences on the surfaces of the macrophytes *C. nodosa* (mixed settlement) and *C. cylindracea* (mixed and monospecific settlement) and in the ambient seawater. The proportion of alphaproteobacterial sequences in the total bacterial and archaeal community is given above the corresponding bar. NR – No Relative (sequences without known relatives within the corresponding group).

**Figure 8:**
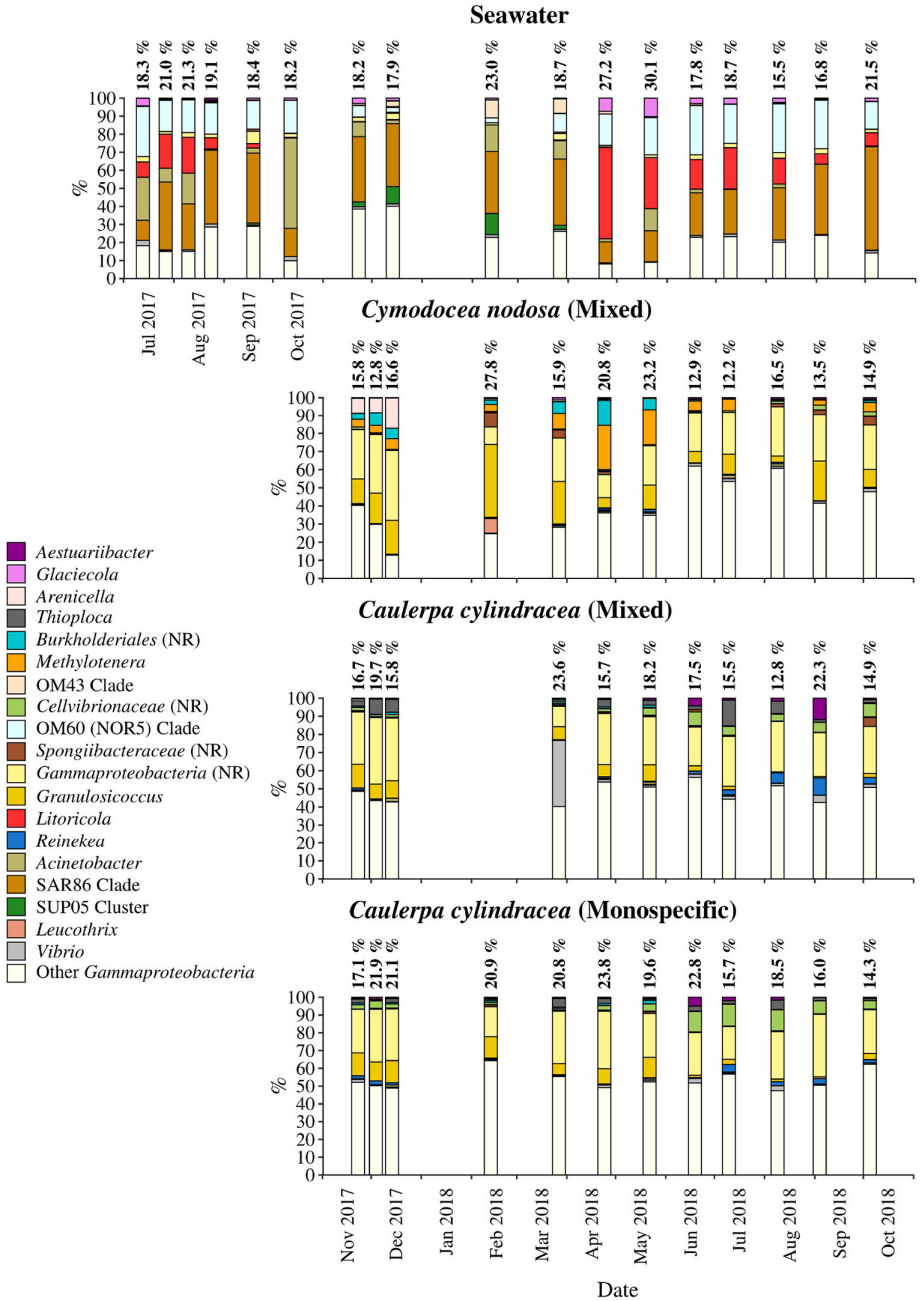
Taxonomic classification and relative contribution of the most abundant (≥1%) gammaproteobacterial sequences on the surfaces of the macrophytes *C. nodosa* (mixed settlement) and *C. cylindracea* (mixed and monospecific settlement) and in the ambient seawater. The proportion of gammaproteobacterial sequences in the total bacterial and archeal community is given above the corresponding bar. NR, no relative (sequences without known relatives within the corresponding group).

**Figure 9:**
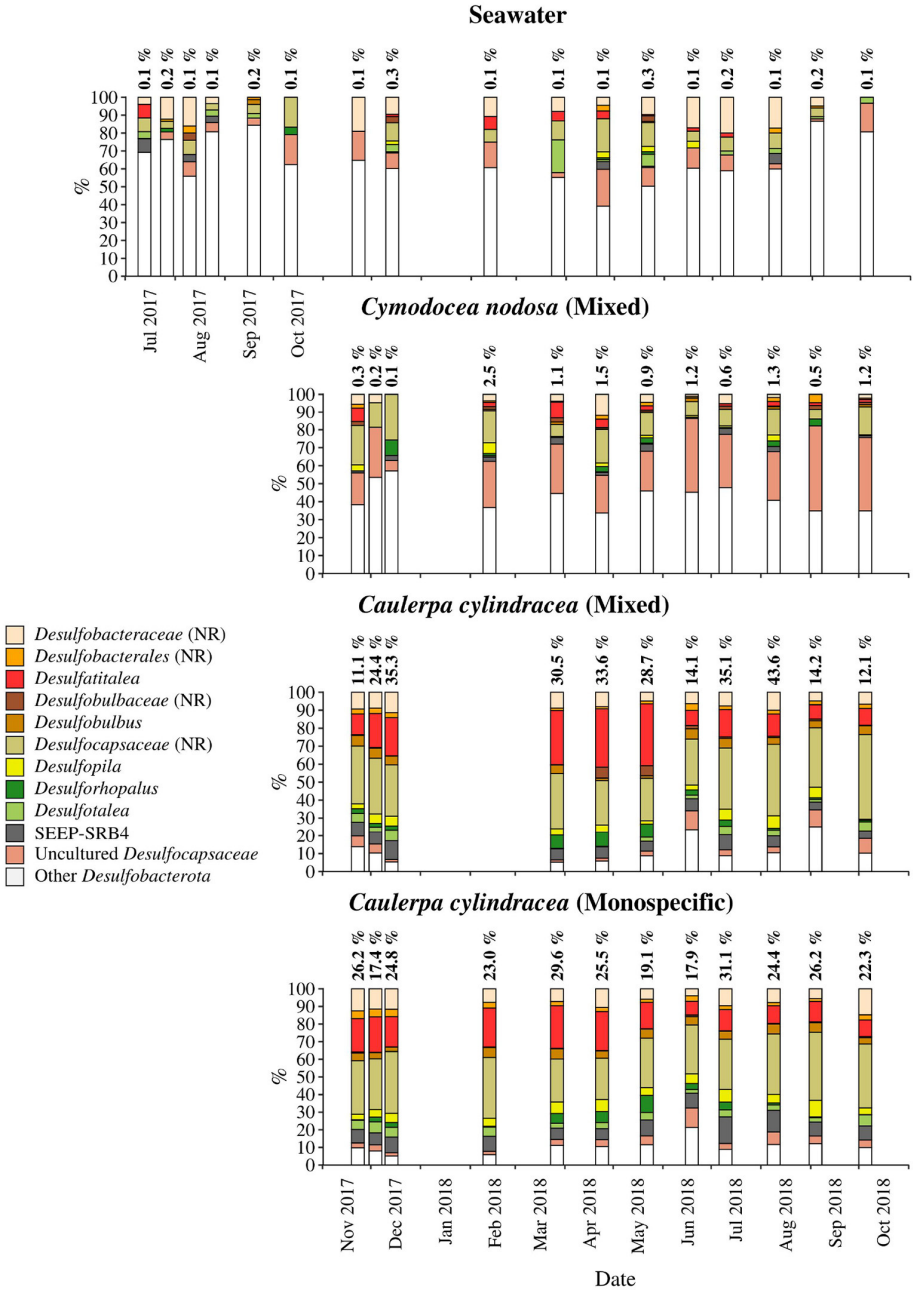
Taxonomic classification and relative contribution of the most abundant (≥ 1%) sequences within the *Desulfobacterota* on the surfaces of the macrophytes *C. nodosa* (mixed settlement) and *C. cylindracea* (mixed and monospecific settlement) and in the ambient seawater. The proportion of sequences classified as *Desulfobacterota* in the total bacterial and archaeal community is given above the corresponding bar. NR, no relative (sequences without known relatives within the corresponding group).

The taxonomic composition of both, macrophyte associated and ambient seawater community, was dominated by bacterial (99.1 ± 2.1%) over archeal sequences (0.9 ± 2.1%; Figure 4). Higher relative abundances of chloroplast related sequences were only observed in surface associated communities, with higher values in autumn/winter (37.2 ± 11.2%) than in spring/summer (20.9 ± 9.7%) (*p* < 0.0001; Supplementary Figure 3). Generally, at higher taxonomic ranks (phylum-class), epiphytic and ambient seawater microbial communities were composed of similar bacterial taxa. Ambient seawater communities were mainly comprised of *Actinobacteriota, Bacteroidota, Cyanobacteria, Alphaproteobacteria, Gammaproteobacteria*, and *Verrucomicrobiota*. Communities associated with *C. nodosa* consisted additionally of *Planctomycetota* contributing more in summer 2018 than in other seasons. In addition, communities from mixed and monospecific *C. cylindracea* were similar and characterized by the same groups as ambient seawater and *C. nodosa* communities with the addition of *Desulfobacterota* (Figure 4). Larger differences between environments and host species were observed at lower taxonomic ranks (Figures 5–9).

*Cyanobacteria* related sequences comprised, on average, 5.5 ± 4.4% of total sequences (Figure 5). Higher proportions were found for *C. nodosa* (16.4 ± 5.3%) and *C. cylindracea* mixed (7.7 ± 3.9%) and monospecific (7.8 ± 2.4%) associated communities in autumn (*p* < 0.0001) and for ambient seawater communities in winter (8.8 ± 7.5%). Large taxonomic differences between surface associated and ambient seawater cyanobacterial communities were observed. Ambient seawater communities were mainly comprised of *Cyanobium* and *Synechococcus*, while surface associated communities were comprised of *Pleurocapsa* and sequences within the class *Cyanobacteriia* that could not be further classified (no relative *Cyanobacteriia*; Figure 5). In addition, seasonal changes in surface associated cyanobacterial communities were observed with *Pleurocapsa* and no relative *Cyanobacteriia* comprising larger proportions of *Cyanobacteria* in autumn and winter and *Acrophormium, Phormidesmis* and sequences without known relatives within the *Nodosilineaceae* (no relative *Nodosilineaceae*) in spring and summer (Figure 5).

Sequences classified as *Bacteroidota* comprised, on average, 19.2 ± 5.5% of all sequences (Figure 6). Similar to *Cyanobacteria*, large differences in the taxonomic composition between ambient seawater and surface associated communities were found (Figure 6). The ambient seawater community was characterized by the NS4 and NS5 marine groups, uncultured *Cryomorphaceae*, uncultured *Flavobacteriaceae*, NS11-12 marine group, *Balneola*, uncultured *Balneolaceae* and *Formosa*. In contrast, in surface associated communities *Lewinella, Portibacter, Rubidimonas*, sequences without known relatives within the *Saprospiraceae* (no relative *Saprospiraceae*), uncultured *Saprospiraceae*, sequences without known relatives within the *Flavobacteriaceae* (no relative *Flavobacteriaceae*) and uncultured *Rhodothermaceae* were found. Some groups showed minor seasonal changes such as no relative *Flavobacteriaceae* whose sequences were more abundant from November 2017 until June 2018. In contrast, uncultured *Rhodothermaceae* showed higher proportions from June 2018 until the end of the study period. Surface associated *Bacteroidota* communities were very diverse as observed in the high proportion of taxa clustering as other *Bacteroidota* (Figure 6).

On average, *Alphaproteobacteria* were in comparison to the other high rank taxa the largest taxonomic group, comprising 29.2 ± 12.0% of all sequences (Figure 8). In accordance to the above described taxa, large differences between ambient seawater and surface associated communities were observed. Ambient seawater communities were composed mainly of the SAR11 clade, AEGEAN-169 marine group, SAR116 clade, sequences without known relatives within the *Rhodobacteraceae* (no relative *Rhodobacteraceae*), HIMB11 and the OCS116 clade, while surface associated communities were composed mainly of no relative *Rhodobacteraceae* and to a lesser degree of *Pseudoahrensia, Amylibacter*, and sequences without known relatives within the *Alphaproteobacteria* (no relative *Alphaproteobacteria*) and *Hyphomonadaceae* (no relative *Hyphomonadaceae*). Representatives of no relative *Rhodobacteraceae* comprised on average 54.7 ± 11.5% of all alphaproteobacterial sequences in the epiphytic community (Figure 7). In addition, *Amylibacter* was detected mainly in *C. nodosa* from November 2017 until March 2018.

Sequences related to *Gammaproteobacteria* comprised on average 18.6 ± 3.9 % of all sequences (Figure 8). Similar to above mentioned taxa, large taxonomic differences between ambient seawater and surface associated communities were found. Ambient seawater communities were mainly comprised of the OM60 (NOR5) clade, *Litoricola, Acinetobacter* and the SAR86 clade, while epiphytic communities were mainly composed of sequences without known relatives within the *Gammaproteobacteria* (no relative *Gammaproteobacteria*) and *Granulosicoccus*. Beside these two groups specific to all three epiphytic communities, *C. nodosa* was characterized by *Arenicella, Methylotenera*, and sequences without known relatives within the *Burkholderiales* (no relative *Burkholderiales*), while *Thioploca, Reinekea*, and sequences without known relatives within *Cellvibrionaceae* (no relative *Cellvibrionaceae*) were more specific to both mixed and monospecific *C. cylindracea*. In addition, *Arenicella* was more pronounced in November and December 2017, while no relative *Burkholderiales* and *Methylotenera* were characteristic for the period from March until May 2018. For the *C. cylindracea* specific taxa no relative *Cellvibrionaceae* and *Reinekea* showed seasonality and were characteristic for samples originating from June to October 2018. In addition, similar to *Bacteroidota*, a large proportion of the surface associated community was grouped as other *Gammaproteobacteria* indicating high diversity within this group (Figure 8).

*Desulfobacterota* were specific for *C. cylindracea*. In the mixed and monospecific *C. cylindracea* communities the proportion of *Desulfobacterota* was 25.7 ± 11.2 and 24.0 ± 4.3%, respectively (Figure 9). In contrast, in ambient seawater and *C. nodosa* communities the contribution of *Desulfobacterota* was only 0.1 ± 0.08 and 1.0 ± 0.7%, respectively. In *C. cylindracea* the community consisted mainly of *Desulfatitalea, Desulfobulbus, Desulfopila, Desulforhopalus, Desulfotalea*, SEEP-SRB4, uncultured *Desulfocapsaceae* and sequences without known relatives within the *Desulfobacteraceae* (no relative *Desulfobacteraceae*), *Desulfobulbaceae* (no relative *Desulfobulbaceae*), and *Desulfocapsaceae* (no relative *Desulfocapsaceae*; Figure 9).

## 4. Discussion

In the present study, we applied a selective epiphytic DNA isolation procedure based on direct cellular lysis (Korlević et al., 2021) coupled with a monthly sampling and Illumina amplicon sequencing to describe in detail the bacterial and archaeal communities associated with the surfaces of two marine macrophytes, *C. nodosa* and *C. cylindracea*. Highest richness was observed for *C. cylindracea* (mixed and monospecific) followed by *C. nodosa* and lowest richness was found in ambient seawater microbial communities. Higher richness of microbial communities associated with macrophytes than in ambient seawater has been described earlier (Mancuso et al., 2016; Ugarelli et al., 2019) and could be attributed to a larger set of inhabitable microniches existing on macrophyte surfaces than in the ambient seawater. The highest richness observed for *C. cylindracea* might be partly due to its contact with the sediment. The stolon of *C. cylindracea* is attached to the sediment surface with rhizoids and thus, the stolon and rhizoids are in direct contact with the sediment. Also, studies have shown that the presence of *C. cylindracea* can alter the content and biochemical composition of sedimentary organic matter (Pusceddu et al., 2016; Rizzo et al., 2017, 2020) possibly further expanding the number of inhabitable microniches and thus causing the observed increase in richness. Seasonal differences in richness observed for surface attached communities indicated a slightly higher richness in spring and summer. This pattern could be explained by a more intense macrophyte growth in these two seasons than in autumn and winter (Zavodnik et al., 1998; Ruitton et al., 2005; Najdek et al., 2020). During their main growth season in spring and summer macrophytes exhibit a more dynamic chemical interaction with the surface community probably causing an increase in the number of inhabitable microniches (Borges and Champenois, 2015; Rickert et al., 2016). Proportions of shared epiphytic OTUs between consecutive sampling points were low also indicated by the proportion of OTUs ( ≤ 1.0%) present at every sampling date (Figure 3). These persistent OTUs, however, accounted for a high proportion of sequences (≥40.2%), as is often the case with similar high-frequency sampling studies (Gilbert et al., 2009, 2012). In comparison to the seawater community, higher values of shared OTUs between consecutive sampling points were observed for the macrophyte surface associated communities. It appears that macrophyte surfaces are providing more stable conditions than the ambient seawater.

We observed a strong differentiation between the surface attached and ambient seawater communities at the level of OTUs which is in agreement with most published studies (Burke et al., 2011b; Michelou et al., 2013; Mancuso et al., 2016; Roth-Schulze et al., 2016; Crump et al., 2018; Ugarelli et al., 2019; Sanders-Smith et al., 2020). This indicates that marine macrophytes are selecting microorganisms from the pool of microbial taxa present in the ambient seawater, modifying the microbial community once the macrophyte associated microbial biofilm develops (Salaün et al., 2012; Michelou et al., 2013). In addition, similar to the study of Roth-Schulze et al. (2016) seagrass and macroalgae specific microbial communities were identified, while no difference between *C. cylindracea* settlements was observed indicating that seagrass and macroalgae specific metabolism is involved in the selection and development of the associated biofilm. At the level of OTUs seasonal changes of *C. nodosa* and *C. cylindracea* associated communities were identified that could be linked to the growth cycle of the seagrass and macroalgae (Agostini et al., 2003; Najdek et al., 2020). *C. nodosa* was characterized by a spring community during maximum seagrass proliferation, a summer community during the highest standing stock of *C. nodosa* and an autumn/winter community during the decay of seagrass biomass. In contrast, *C. cylindracea* started to proliferate in late spring and was characterized only by a summer community during high growth rates and by an autumn/winter/spring community when the biomass was at the peak and decaying thereafter. Similar seasonal changes in the epiphytic community have also been described for other macroalgae (Tujula et al., 2010; Lachnit et al., 2011).

The taxonomic analysis showed higher chloroplast sequence abundances in autumn/winter than in spring/summer. This pattern is not surprising as seagrasses harbour more algal epiphytes during autumn/winter than in spring/summer (Reyes and Sansón, 2001). Furthermore, we used an adapted DNA isolation protocol that is known to partially co-extract DNA from planktonic eukaryotes (Korlević et al., 2015). In general, the taxonomic analysis identified epiphytic phylogenetic groups present throughout the year comprising most of the reads, and taxa present in lower proportions showing seasonal patterns. The first group was comprised of members of the *Bacteroidota* family *Saprospiraceae*, the alphaproteobacterial *Rhodobacteraceae* and *Hyphomonadaceae*, the gammaproteobacterial genus *Granulosicoccus*, sequences without known relatives within *Gammaproteobacteria* and various taxa within *Desulfobacterota* (Figures 6–9). All these groups were found on all host species, with the exception of *Desulfobacterota* that was characteristic for *C. cylindracea*. In addition, the persistence of *Rhodobacteraceae* in the case of *C. nodosa* and *Desulfobacterota* in the case of *C. cylindracea* could be observed in the taxonomic classification of OTUs present at every sampling date. Within the *Bacteroidota* different groups within *Saprospiraceae* (e.g., *Lewinella, Portibacter*, and *Rubidimonas*) were identified to be persistent. It has been suggested that members of this family are important in the hydrolysis and utilization of complex organic sources (McIlroy and Nielsen, 2014). Surface attached life style would be beneficial to these microbes as they could thrive on products of host cellular breakdown or by-products of host metabolism, so it not surprising that they are often found associated with macrophyte surfaces (Burke et al., 2011b; McIlroy and Nielsen, 2014; Crump et al., 2018). *Rhodobacteraceae* are often detected on macrophyte surfaces and usually are one of the most abundant groups (Burke et al., 2011b; Michelou et al., 2013; Mancuso et al., 2016). The functional association between macrophytes and members of this groups is difficult to assess based on 16S rRNA analysis as this family is phenotypically, metabolically, and ecologically very diverse (Pujalte et al., 2014). However, some interesting metabolic capacities linked to this group were described. Genomic analysis of *Rhodobacteraceae* strains and metatranscriptomic sequencing of seagrass microbiomes revealed the potential for biosynthesis of indole-3-acetic acid (IAA), a plant hormone (Simon et al., 2017), indicating a possible intake by seagrasses. However, another study found no effect of IAA on *C. nodosa* growth showing the complexity of macrophyte–microbes interactions (Muñoz, 1995). Another persistent alphaproteobacterial family was the *Hyphomonadaceae*, a group that contain species with stalks used to attach cells to different surfaces (Abraham and Rohde, 2014). This group has been previously associated with seagrass surfaces (Weidner et al., 2000) and it is believed that possessing stalks could be an advantage to keep the cells in the proximity of exudate excreted by the host (Weidner et al., 2000; Abraham and Rohde, 2014).

Within the *Gammaproteobacteria*, sequences without known representatives were the most pronounced group present throughout the year. *Gammaproteobacteria* are often a major constituent of macrophyte epiphytic communities (Burke et al., 2011b; Michelou et al., 2013; Crump et al., 2018). A study has attributed the expression of enzymes for the degradation of galactose-based algal polymers to this class indicating their possible involvement into epibiotic algal biofilm control (Crump et al., 2018). In addition, *Granulosicoccus* was also found in almost all samples. A species of this genus has been isolated from the leaf surface of the seagrass *Zostera marina* (Kurilenko et al., 2010), while sequences related to this genus have been found on the surfaces of macroalgae (Lachnit et al., 2011; Bengtsson et al., 2012), including *C. cylindracea* (Rizzo et al., 2016a), indicating this group preference for macrophyte surfaces. It is possible that bacteria of this genus can thrive on exudates of different macrophytes as it is known from cultivated members that they can utilize various sugars and amino acids (Ivanova and Webb, 2014). The presence of *Desulfobacterota* only on *C. cylindracea* is to be expected as part of the epiphytic community is in direct contact with the sediment. The *Desulfobacterota* community was comprised of known sulphate sediment groups such as the *Desulfatitalea* and no relative *Desulfocapsaceae* (Kuever, 2014; Higashioka et al., 2015). Sequences related to sulphur cycling bacterial groups have been found in *Caulerpa* endophytic and epiphytic communities (Aires et al., 2013). It is possible that these groups are involved into enhanced sulphate reduction rates observed in sediments underlying *Caulerpa* settlements causing unsuitable conditions to sulphide-sensitive seagrasses (Holmer et al., 2009).

The only high rank taxonomic group showing strong seasonal fluctuations was *Cyanobacteria*. Cyanobacterial sequences were more pronounced in November and December than in spring and summer. In the months of high cyanobacterial sequence abundances the majority of sequences from this group were classified as *Pleurocapsa*, a group known to colonize different living and non-living surfaces (Burns et al., 2004; Longford et al., 2007; Mobberley et al., 2012; Reisser et al., 2014; Kolda et al., 2020). While we observed a strong temporal pattern for this group, a study of surface sediment cyanobacterial communities did not find any seasonal dynamics for *Pleurocapsa* (Kolda et al., 2020), indicating a possibility that there is a reduced selection of the epiphytic community by the seagrass during periods of low photosynthetic activity (Zavodnik et al., 1998), causing leaves to become a suitable surface for non-specific colonizers. Beside all these groups comprising most of the sequences, a set of taxa present in lower proportions and showing seasonal patterns was identified. This group was comprised of e.g., *Bacteroidota* sequences without known relatives within *Flavobacteriaceae* and *Rhodothermaceae*, the alphaproteobacterial *Amylibacter* and the gammaproteobacterial *Methylotenera, Reinekea* and sequences without known relatives within *Cellvibrionaceae* (Figures 6, 8).

It is possible that *Flavobacteriaceae* and *Rhodothermaceae* are occupying similar niches with *Rhodothermaceae* being more adapted to higher temperatures as it is known that culturable members of this family exhibit mesophilic and thermophilic characteristics (Park et al., 2014). This would explain why we observed a higher presence of *Rhodothermaceae* in the warmer period of the year. A strain belonging to the *Rhodobacteraceae* genus *Amylibacter* has been isolated from the surface of a green macroalga indicating that members of this group can exhibit surface attached life style (Nedashkovskaya et al., 2016). In addition, since this is a relatively novel genus it is possible that novel taxa within *Rhodobacteraceae* will be described in the future elucidating the taxonomy of the currently high proportion of *Rhodobacteraceae* sequences without known relatives. The genus *Methylotenera* belongs to the methylotrophic family *Methylophilaceae*, a group capable of oxidizing non-methane single-carbon compounds such as methanol and methylamine (Chistoserdova and Kalyuzhnaya, 2018). Interestingly, angiosperms produce methanol during cell-wall synthesis (Nemecek-Marshall et al., 1995; Dorokhov et al., 2018), so it is not surprising that we found members of this genus only on *C. nodosa* and in spring, during a period of maximum seagrass proliferation. Other studies have also found *Methylotenera*-specific sequences associated with seagrass roots and leaves indicating that this group members are important constituents of the seagrass microbiome (Crump et al., 2018; Sanders-Smith et al., 2020). Genomic and physiological analyses of cultivated *Cellvibrionaceae* and *Reinekea* members showed the capabilities to use important algal polysaccharides (Avcı et al., 2017; Xie et al., 2017) indicating their possible involvement into the degradation of *C. cylindracea* polysaccharides and/or the control of its algal epiphytes.

The epiphytic microbial community associated with marine macrophytes is undergoing seasonal changes that can be attributed to the fluctuations of environmental conditions, the growth cycle of macrophytes inhabiting temperate zones or the combined effect of both. In the present study, we could identify in analyzed high rank taxa phylogenetic groups present throughout the year, comprising most of the sequences and a lower proportion of taxa showing seasonal patterns connected to the macrophyte growth cycle (Figures 4, 9). Studies focusing on functional comparisons between communities associated with different hosts showed that the majority of functions could be found in every community, indicating functional redundancy (Roth-Schulze et al., 2016). This difference between phylogenetic variability and functional stability has been explained by the lottery hypothesis assuming an initial random colonization step performed by a set of functionally equivalent taxonomic groups (Burke et al., 2011a; Roth-Schulze et al., 2016). It is possible that functional redundancy is a characteristic of high abundance taxa detected to be present throughout the year, while seasonal and/or host-specific functions are an attribute of taxa displaying successional patterns. Further studies connecting taxonomy with functional properties will be required to elucidate the degree of functional redundancy or specificity in epiphytic microbial communities.

## Data Availability Statement

The datasets presented in this study can be found in online repositories. The names of the repositories and the accession number can be found in the article.

## Author Contributions

MK, GH, and MN designed the study. MK, MM, ZZ, and MN performed sampling and laboratory analysis. MK, MM, and ZZ analyzed the data. MK prepared the manuscript with editorial help from MM, ZZ, GH, and MN. All authors contributed to the article and approved the final submitted version.

## Funding

This work was funded by the Croatian Science Foundation through the MICRO-SEAGRASS project (project number IP-2016-06-7118). ZZ and GH were supported by the Austrian Science Fund (FWF) project ARTEMIS (project number P28781-B21).

## Conflict of Interest

The authors declare that the research was conducted in the absence of any commercial or financial relationships that could be construed as a potential conflict of interest.

## Publisher's Note

All claims expressed in this article are solely those of the authors and do not necessarily represent those of their affiliated organizations, or those of the publisher, the editors and the reviewers. Any product that may be evaluated in this article, or claim that may be made by its manufacturer, is not guaranteed or endorsed by the publisher.
