# Supplementary material for: Seasonal Dynamics of Epiphytic Microbial Communities on Marine Macrophyte Surfaces
Source: Front Microbiol. 2021 Sep 9;12:671342. doi: 10.3389/fmicb.2021.671342 (PMC8482799; doi:10.3389/fmicb.2021.671342)
Supplement: Supplementary file 1 [file Data_Sheet_1.pdf]

Supplementary Material

Seasonal Dynamics of Epiphytic Microbial Communities on Marine Macrophyte Surfaces

Marino Korlević[1*], Marsej Markovski[1], Zihao Zhao[2], Gerhard J. Herndl[2,3], Mirjana Najdek[1]

1. Center for Marine Research, Ruđer Bošković Institute, Croatia

2. Department of Functional and Evolutionary Ecology, University of Vienna, Austria

3. Department of Marine Microbiology and Biogeochemistry, Royal Netherlands Institute for Sea Research (NIOZ), Utrecht University, The Netherlands

*To whom correspondence should be addressed:

Marino Korlević

G. Paliaga 5, 52210 Rovinj, Croatia

Tel.: +385 52 804 768

Fax: +385 52 804 780

e-mail: marino.korlevic@irb.hr

Running title: Seasonal dynamics of epiphytic communities

Supplementary figures

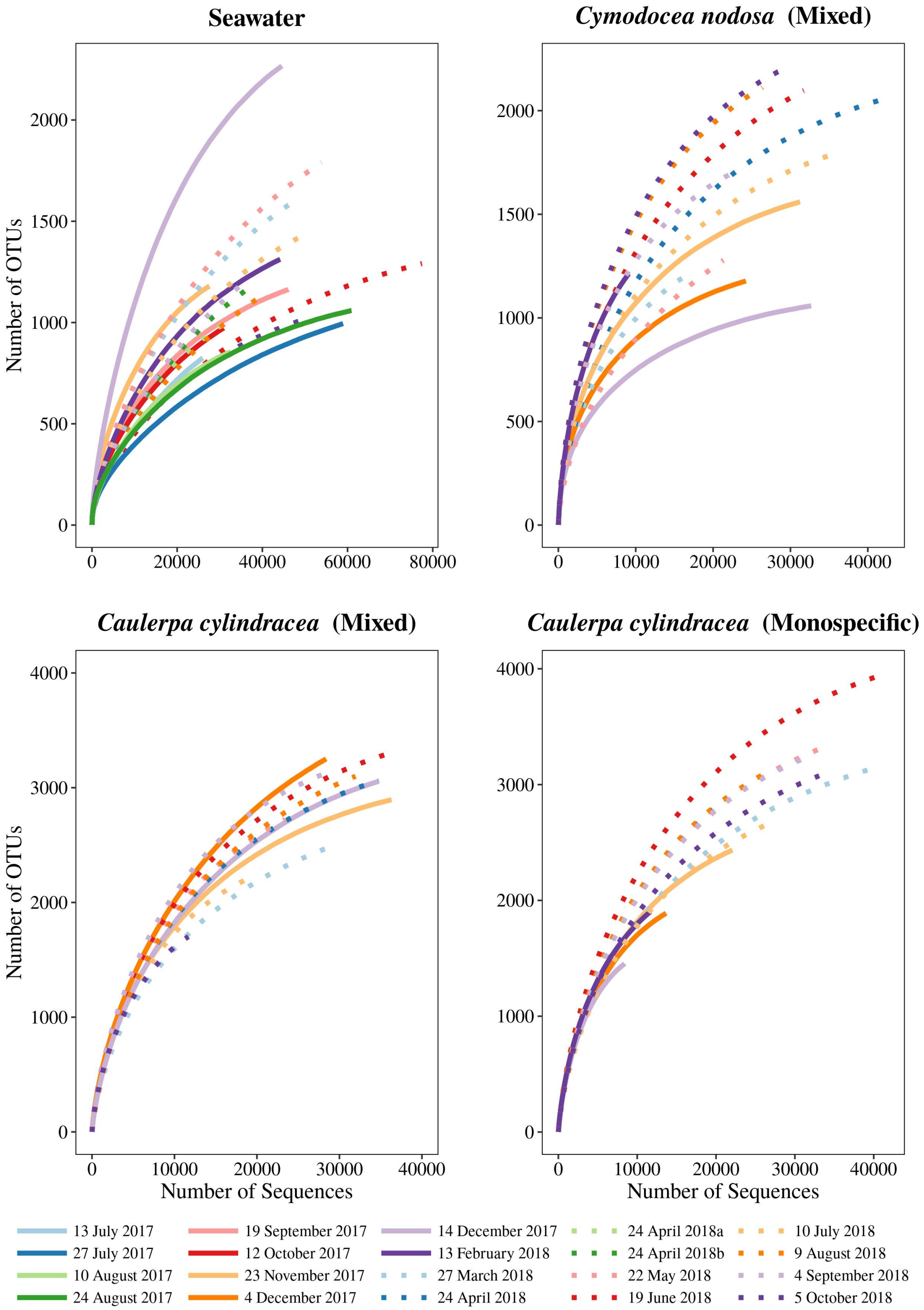

Figure S1. Rarefaction curves of bacterial and archaeal communities from the surfaces of the macrophytes *C. nodosa* (mixed settlement) and *C. cylindracea* (mixed and monospecific settlement) and in the ambient seawater.

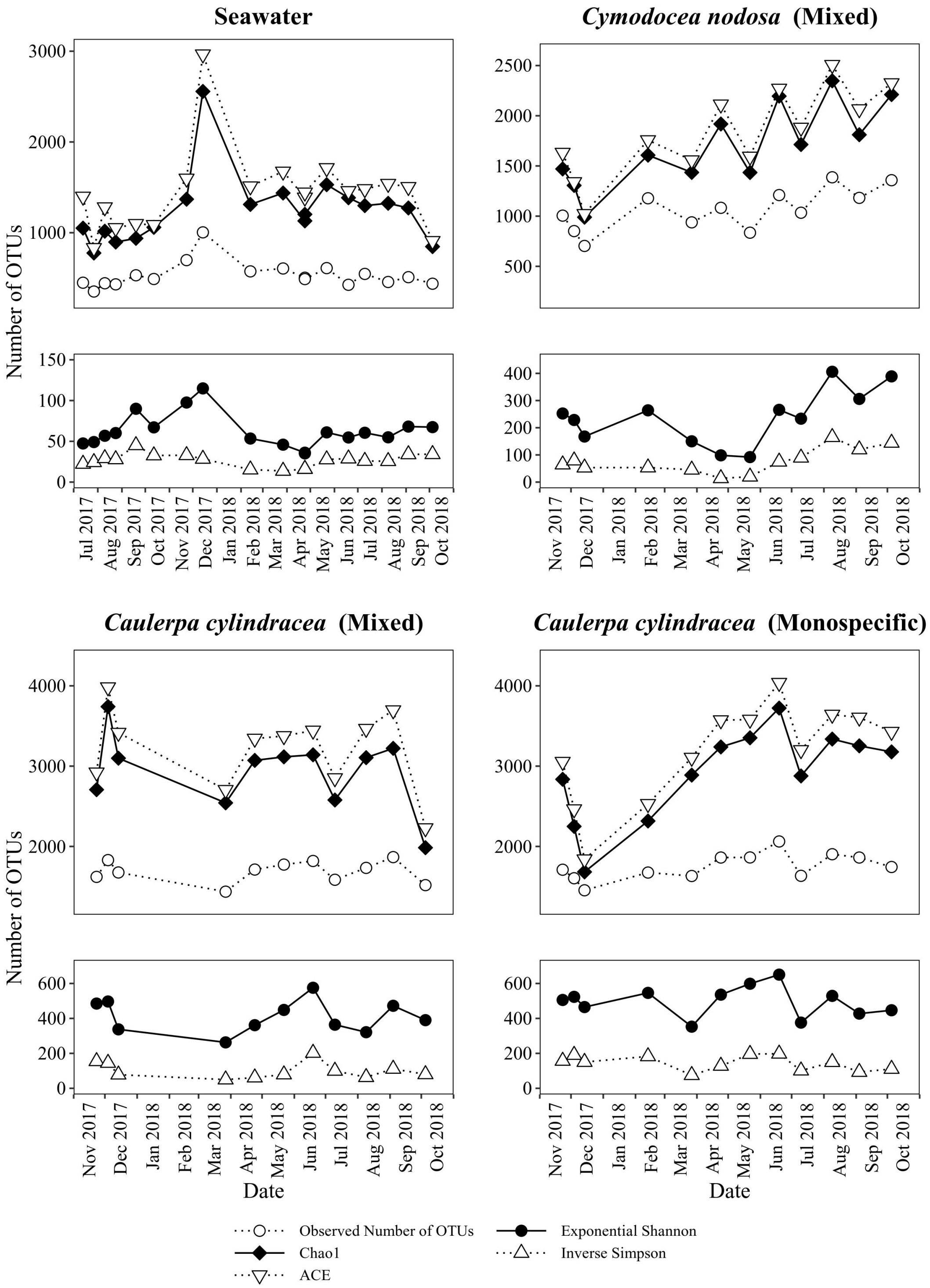

Figure S2. Seasonal dynamics of observed number of OTUs, Chao1, ACE, exponential of the Shannon diversity index and Inverse Simpson index of bacterial and archaeal communities from the surfaces of the macrophytes *C. nodosa* (mixed settlement) and *C. cylindracea* (mixed and monospecific settlement) and in the ambient seawater.

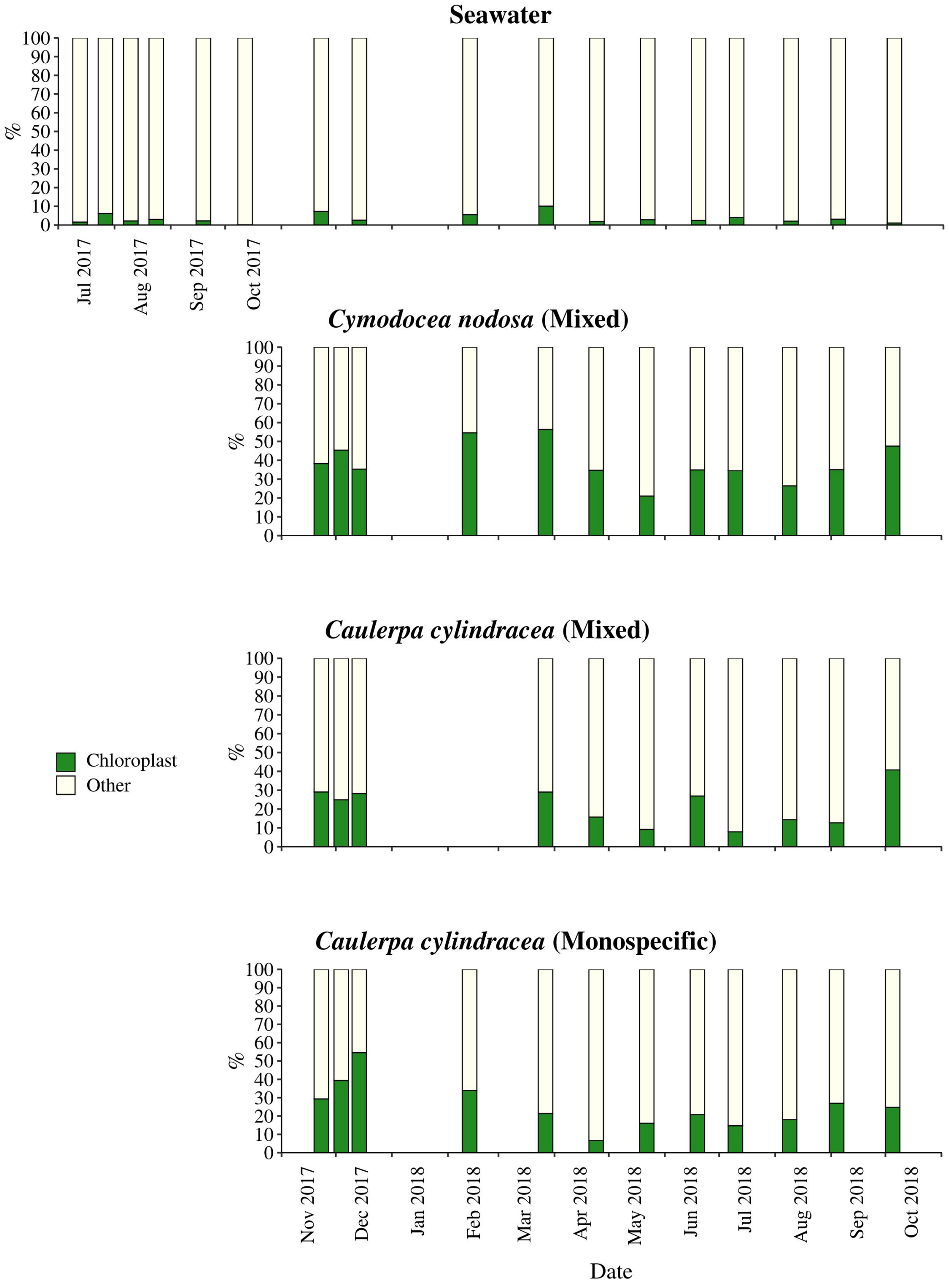

Figure S3. Relative contribution of chloroplast sequences on the surfaces of the macrophytes *C. nodosa* (mixed settlement) and *C. cylindracea* (mixed and monospecific settlement) and in the ambient seawater.

Supplementary table

Table S1. Sample ID, community type, sampling date and season, no. of sequences and no. of OTUs of each sample. The number of sequences and OTUs was calculated after exclusion of sequences without known relatives (no relative sequences) and eukaryotic, chloroplast and mitochondrial sequences.

Sample ID	Community Type	Date	Season	No. of Sequences	No. of OTUs
3	Seawater	13 July 2017	Summer	26,006	825
5	Seawater	27 July 2017	Summer	58,951	995
7	Seawater	10 August 2017	Summer	32,623	856
9	Seawater	24 August 2017	Summer	60,938	1,059
11	Seawater	19 September 2017	Summer	46,106	1,163
13	Seawater	12 October 2017	Autumn	30,905	973
15	Seawater	23 November 2017	Autumn	27,582	1,180
17	Seawater	14 December 2017	Autumn	44,591	2,267
19	Seawater	13 February 2018	Winter	44,193	1,312
21	Seawater	27 March 2018	Winter	46,352	1,583
23a	Seawater	24 April 2018	Spring	30,976	995
23b	Seawater	24 April 2018	Spring	38,565	1,196
25	Seawater	22 May 2018	Spring	53,872	1,791
27	Seawater	19 June 2018	Spring	77,463	1,291
29	Seawater	10 July 2018	Summer	50,786	1,443
31	Seawater	9 August 2018	Summer	39,039	1,120
33	Seawater	4 September 2018	Summer	36,201	1,205
35	Seawater	5 October 2018	Autumn	49,585	1,014
37	*Cymodocea nodosa* (Mixed)	23 November 2017	Autumn	31,241	1,560
41	*Cymodocea nodosa* (Mixed)	4 December 2017	Autumn	24,241	1,178
45	*Cymodocea nodosa* (Mixed)	14 December 2017	Autumn	32,686	1,058
49	*Cymodocea nodosa* (Mixed)	13 February 2018	Winter	9,091	1,213
52	*Cymodocea nodosa* (Mixed)	27 March 2018	Winter	17,000	1,215
55	*Cymodocea nodosa* (Mixed)	24 April 2018	Spring	42,653	2,063
58	*Cymodocea nodosa* (Mixed)	22 May 2018	Spring	21,337	1,278
61	*Cymodocea nodosa* (Mixed)	19 June 2018	Spring	31,726	2,097
64	*Cymodocea nodosa* (Mixed)	10 July 2018	Summer	35,746	1,793
67	*Cymodocea nodosa* (Mixed)	9 August 2018	Summer	26,360	2,113

Table S1. Sample ID, community type, sampling date and season, no. of sequences and no. of OTUs of each sample. The number of sequences and OTUs was calculated after exclusion of sequences without known relatives (no relative sequences) and eukaryotic, chloroplast and mitochondrial sequences. *(continued)*

Sample ID	Community Type	Date	Season	No. of Sequences	No. of OTUs
70	*Cymodocea nodosa* (Mixed)	4 September 2018	Summer	23,276	1,713
73	*Cymodocea nodosa* (Mixed)	5 October 2018	Autumn	29,910	2,216
38	*Caulerpa cylindracea* (Mixed)	23 November 2017	Autumn	36,318	2,895
42	*Caulerpa cylindracea* (Mixed)	4 December 2017	Autumn	28,388	3,251
46	*Caulerpa cylindracea* (Mixed)	14 December 2017	Autumn	34,721	3,055
53	*Caulerpa cylindracea* (Mixed)	27 March 2018	Winter	28,688	2,478
56	*Caulerpa cylindracea* (Mixed)	24 April 2018	Spring	34,765	3,060
59	*Caulerpa cylindracea* (Mixed)	22 May 2018	Spring	23,403	2,723
62	*Caulerpa cylindracea* (Mixed)	19 June 2018	Spring	36,487	3,310
65	*Caulerpa cylindracea* (Mixed)	10 July 2018	Summer	18,486	2,192
68	*Caulerpa cylindracea* (Mixed)	9 August 2018	Summer	31,953	3,099
71	*Caulerpa cylindracea* (Mixed)	4 September 2018	Summer	29,280	3,152
74	*Caulerpa cylindracea* (Mixed)	5 October 2018	Autumn	11,698	1,702
39	*Caulerpa cylindracea* (Monospecific)	23 November 2017	Autumn	22,086	2,435
43	*Caulerpa cylindracea* (Monospecific)	4 December 2017	Autumn	13,661	1,890
47	*Caulerpa cylindracea* (Monospecific)	14 December 2017	Autumn	8,408	1,454
51	*Caulerpa cylindracea* (Monospecific)	13 February 2018	Winter	11,673	1,902
54	*Caulerpa cylindracea* (Monospecific)	27 March 2018	Winter	39,469	3,131
57	*Caulerpa cylindracea* (Monospecific)	24 April 2018	Spring	20,299	2,832
60	*Caulerpa cylindracea* (Monospecific)	22 May 2018	Spring	33,042	3,305
63	*Caulerpa cylindracea* (Monospecific)	19 June 2018	Spring	41,852	3,964
66	*Caulerpa cylindracea* (Monospecific)	10 July 2018	Summer	27,036	2,673
69	*Caulerpa cylindracea* (Monospecific)	9 August 2018	Summer	26,736	3,114
72	*Caulerpa cylindracea* (Monospecific)	4 September 2018	Summer	31,872	3,246
75	*Caulerpa cylindracea* (Monospecific)	5 October 2018	Autumn	33,086	3,076

Table S2. Richness estimators and diversity indices of each community type. Calculations were done after normalization to the minimum number of reads per sample.

Community Type	Observed No. of OTUs	Chao1	ACE	Exponential Shannon	Inverse Simpson
Seawater	531.0 ± 143.9	1,244.5 ± 392.7	1,441.1 ± 458.7	62.2 ± 20.5	26.7 ± 8.0
Cymodocea nodosa (Mixed)	1,063.7 ± 210.6	1,703.2 ± 409.9	1,838.8 ± 437.3	237.8 ± 100.4	76.9 ± 46.6
Caulerpa cylindracea (Mixed)	1,688.4 ± 136.6	2,936.9 ± 460.2	3,220.7 ± 497.2	410.4 ± 92.5	102.2 ± 47.2
Caulerpa cylindracea (Monospecific)	1,750.4 ± 165.7	2,911.0 ± 575.0	3,171.7 ± 622.1	496.4 ± 87.1	144.4 ± 42.6

Table S3. Statistic of richness estimator and diversity index parameters for different community types. Parameters were tested by applying the Kruskal-Wallis *H* test followed by a pairwise comparison using the Mann-Whitney *U* test. Bonferroni correction was used to address the problem of multiple comparisons.

	**Kruskal-Wallis *H* test**			**Mann-Whitney *U* test**	
**Parameter**	***H***	***df***	***p***	**Comparisons Between Community Types**	***p***
Observed No. of OTUs	44.8	3	< 0.0001	Seawater – *Cymodocea nodosa* (Mixed)	< 0.0001
				Seawater – *Caulerpa cylindracea* (Mixed)	< 0.0001
				Seawater – *Caulerpa cylindracea* (Monospecific)	< 0.0001
				Cymodocea nodosa (Mixed) – *Caulerpa cylindracea* (Mixed)	< 0.001
				Cymodocea nodosa (Mixed) – *Caulerpa cylindracea* (Monospecific)	< 0.001
				Caulerpa cylindracea (Mixed) – *Caulerpa cylindracea* (Monospecific)	1.00
Chao1	38.3	3	< 0.0001	Seawater – *Cymodocea nodosa* (Mixed)	< 0.01
				Seawater – *Caulerpa cylindracea* (Mixed)	< 0.0001
				Seawater – *Caulerpa cylindracea* (Monospecific)	< 0.0001
				Cymodocea nodosa (Mixed) – *Caulerpa cylindracea* (Mixed)	< 0.0001
				Cymodocea nodosa (Mixed) – *Caulerpa cylindracea* (Monospecific)	< 0.001
				Caulerpa cylindracea (Mixed) – *Caulerpa cylindracea* (Monospecific)	1.00
ACE	37.1	3	< 0.0001	Seawater – *Cymodocea nodosa* (Mixed)	< 0.05
				Seawater – *Caulerpa cylindracea* (Mixed)	< 0.0001
				Seawater – *Caulerpa cylindracea* (Monospecific)	< 0.0001
				Cymodocea nodosa (Mixed) – *Caulerpa cylindracea* (Mixed)	< 0.0001
				Cymodocea nodosa (Mixed) – *Caulerpa cylindracea* (Monospecific)	< 0.001
				Caulerpa cylindracea (Mixed) – *Caulerpa cylindracea* (Monospecific)	1.00
Exponential Shannon	43.3	3	< 0.0001	Seawater – *Cymodocea nodosa* (Mixed)	< 0.0001
				Seawater – *Caulerpa cylindracea* (Mixed)	< 0.0001
				Seawater – *Caulerpa cylindracea* (Monospecific)	< 0.0001
				Cymodocea nodosa (Mixed) – *Caulerpa cylindracea* (Mixed)	< 0.01
				Cymodocea nodosa (Mixed) – *Caulerpa cylindracea* (Monospecific)	< 0.0001
				Caulerpa cylindracea (Mixed) – *Caulerpa cylindracea* (Monospecific)	0.26

Table S3. Statistic of richness estimator and diversity index parameters for different community types. Parameters were tested by applying the Kruskal-Wallis *H* test followed by a pairwise comparison using the Mann-Whitney *U* test. Bonferroni correction was used to address the problem of multiple comparisons. *(continued)*

	**Kruskal-Wallis *H* test**			**Mann-Whitney *U* test**	
**Parameter**	***H***	***df***	***p***	**Comparisons Between Community Types**	***p***
Inverse Simpson	34.8	3	< 0.0001	Seawater – *Cymodocea nodosa* (Mixed)	< 0.01
				Seawater – *Caulerpa cylindracea* (Mixed)	< 0.0001
				Seawater – *Caulerpa cylindracea* (Monospecific)	< 0.0001
				Cymodocea nodosa (Mixed) – *Caulerpa cylindracea* (Mixed)	1.00
				Cymodocea nodosa (Mixed) – *Caulerpa cylindracea* (Monospecific)	< 0.01
				Caulerpa cylindracea (Mixed) – *Caulerpa cylindracea* (Monospecific)	0.26

Table S4. Taxonomic classification of OTUs present at every sampling date in each community type. Only ten OTUs with the highest number of sequences after normalization to the minimum number of reads per sample are shown. NR – No Relative (sequences without known relatives within the corresponding group)

Community Type	OTU Number	No. of Sequences	OTU Taxonomy
Cymodocea nodosa (Mixed)	Otu00014	5092	*Bacteria*; *Proteobacteria*; *Alphaproteobacteria*; *Rhodobacterales*; *Rhodobacteraceae*; *Rhodobacteraceae* (NR)
	Otu00020	3188	*Bacteria*; *Proteobacteria*; *Alphaproteobacteria*; *Rhodobacterales*; *Rhodobacteraceae*; *Rhodobacteraceae* (NR)
	Otu00019	3026	*Bacteria*; *Cyanobacteria*; *Cyanobacteriia*; *Cyanobacteriales*; *Xenococcaceae*; *Pleurocapsa*
	Otu00030	2014	*Bacteria*; *Proteobacteria*; *Alphaproteobacteria*; *Rhodobacterales*; *Rhodobacteraceae*; *Rhodobacteraceae* (NR)
	Otu00013	1884	*Bacteria*; *Proteobacteria*; *Alphaproteobacteria*; *Rhodobacterales*; *Rhodobacteraceae*; *Rhodobacteraceae* (NR)
	Otu00032	1586	*Bacteria*; *Verrucomicrobiota*; *Verrucomicrobiae*; *Opitutales*; *Puniceicoccaceae*; *Lentimonas*
	Otu00059	1469	*Bacteria*; *Proteobacteria*; *Gammaproteobacteria*; *Burkholderiales*; *Methylophilaceae*; *Methylotenera*
	Otu00005	1337	*Bacteria*; *Proteobacteria*; *Alphaproteobacteria*; *Rhodobacterales*; *Rhodobacteraceae*; *Rhodobacteraceae* (NR)
	Otu00050	1264	*Bacteria*; *Proteobacteria*; *Alphaproteobacteria*; *Rhodobacterales*; *Rhodobacteraceae*; *Rhodobacteraceae* (NR)
	Otu00029	1162	*Bacteria*; *Proteobacteria*; *Alphaproteobacteria*; *Rhodobacterales*; *Rhodobacteraceae*; *Rhodobacteraceae* (NR)
Caulerpa cylindracea (Mixed)	Otu00011	4051	*Bacteria*; *Desulfobacterota*; *Desulfobacteria*; *Desulfobacterales*; *Desulfosarcinaceae*; *Desulfatitalea*
	Otu00010	3436	*Bacteria*; *Desulfobacterota*; *Desulfobulbia*; *Desulfobulbales*; *Desulfocapsaceae*; *Desulfocapsaceae* (NR)
	Otu00013	2531	*Bacteria*; *Proteobacteria*; *Alphaproteobacteria*; *Rhodobacterales*; *Rhodobacteraceae*; *Rhodobacteraceae* (NR)
	Otu00021	1859	*Bacteria*; *Desulfobacterota*; *Desulfobulbia*; *Desulfobulbales*; *Desulfocapsaceae*; *Desulfocapsaceae* (NR)
	Otu00025	1481	*Bacteria*; *Desulfobacterota*; *Desulfobulbia*; *Desulfobulbales*; *Desulfocapsaceae*; SEEP-SRB4
	Otu00037	1209	*Bacteria*; *Desulfobacterota*; *Desulfobulbia*; *Desulfobulbales*; *Desulfobulbaceae*; *Desulfobulbus*
	Otu00033	1189	*Bacteria*; *Desulfobacterota*; *Desulfobulbia*; *Desulfobulbales*; *Desulfocapsaceae*; *Desulfocapsaceae* (NR)
	Otu00044	1042	*Bacteria*; *Desulfobacterota*; *Desulfobulbia*; *Desulfobulbales*; *Desulfocapsaceae*; *Desulfocapsaceae* (NR)
	Otu00019	1012	*Bacteria*; *Cyanobacteria*; *Cyanobacteriia*; *Cyanobacteriales*; *Xenococcaceae*; *Pleurocapsa*
	Otu00046	982	*Bacteria*; *Desulfobacterota*; *Desulfobulbia*; *Desulfobulbales*; *Desulfocapsaceae*; *Desulfotalea*

Table S4. Taxonomic classification of OTUs present at every sampling date in each community type. Only ten OTUs with the highest number of sequences after normalization to the minimum number of reads per sample are shown. NR – No Relative (sequences without known relatives within the corresponding group) *(continued)*

Community Type	OTU Number	No. of Sequences	OTU Taxonomy
Caulerpa cylindracea (Monospecific)	Otu00010	3572	*Bacteria*; *Desulfobacterota*; *Desulfobulbia*; *Desulfobulbales*; *Desulfocapsaceae*; *Desulfocapsaceae* (NR)
	Otu00011	2977	*Bacteria*; *Desulfobacterota*; *Desulfobacteria*; *Desulfobacterales*; *Desulfosarcinaceae*; *Desulfatitalea*
	Otu00013	2109	*Bacteria*; *Proteobacteria*; *Alphaproteobacteria*; *Rhodobacterales*; *Rhodobacteraceae*; *Rhodobacteraceae* (NR)
	Otu00021	1931	*Bacteria*; *Desulfobacterota*; *Desulfobulbia*; *Desulfobulbales*; *Desulfocapsaceae*; *Desulfocapsaceae* (NR)
	Otu00025	1912	*Bacteria*; *Desulfobacterota*; *Desulfobulbia*; *Desulfobulbales*; *Desulfocapsaceae*; SEEP-SRB4
	Otu00033	1613	*Bacteria*; *Desulfobacterota*; *Desulfobulbia*; *Desulfobulbales*; *Desulfocapsaceae*; *Desulfocapsaceae* (NR)
	Otu00040	1289	*Bacteria*; *Desulfobacterota*; *Desulfobacteria*; *Desulfobacterales*; *Desulfobacteraceae*; *Desulfobacteraceae* (NR)
	Otu00046	1089	*Bacteria*; *Desulfobacterota*; *Desulfobulbia*; *Desulfobulbales*; *Desulfocapsaceae*; *Desulfotalea*
	Otu00037	1058	*Bacteria*; *Desulfobacterota*; *Desulfobulbia*; *Desulfobulbales*; *Desulfobulbaceae*; *Desulfobulbus*
	Otu00058	950	*Bacteria*; *Desulfobacterota*; *Desulfobacteria*; *Desulfobacterales*; *Desulfosarcinaceae*; *Desulfatitalea*
